# Body mass index and incidence of lung cancer in the HUNT study: using observational and Mendelian randomization approaches

**DOI:** 10.1186/s12885-022-10215-0

**Published:** 2022-11-08

**Authors:** Lin Jiang, Yi-Qian Sun, Ben Michael Brumpton, Arnulf Langhammer, Yue Chen, Xiao-Mei Mai

**Affiliations:** 1 Department of Public Health and Nursing, Faculty of Medicine and Health Science, Norwegian University of Science and Technology (NTNU), MTFS, N-7491, Postbox 8905 Trondheim, Norway; 2 Department of Clinical and Molecular Medicine, Faculty of Medicine and Health Science, Norwegian University of Science and Technology, Trondheim, Norway; 3 Department of Pathology, Clinic of Laboratory Medicine, St. Olavs Hospital, Trondheim University Hospital, Trondheim, Norway; 4 TkMidt-Center for Oral Health Services and Research, Mid-Norway, Trondheim, Norway; 5 Clinic of Medicine, St. Olavs Hospital, Trondheim University Hospital, Trondheim, Norway; 6 K.G. Jebsen Centre for Genetic Epidemiology, Department of Public Health and Nursing, Norwegian University of Science and Technology, Trondheim, Norway; 7 HUNT Research Centre, Department of Public Health and Nursing, Norwegian University of Science and Technology, Levanger, Norway; 8 Levanger Hospital, Nord-Trøndelag Hospital Trust, Levanger, Norway; 9 School of Epidemiology and Public Health, Faculty of Medicine, University of Ottawa, Ottawa, Canada

**Keywords:** Body mass index, Lung cancer incidence, Multivariable Mendelian randomization, Obesity, Prospective cohort, The HUNT Study

## Abstract

**Background:**

Traditional observational studies have shown an inverse association between body mass index (BMI) and lung cancer risk. Mendelian randomization (MR) analysis using genetic variants as instruments for BMI may clarify the nature of the association.

**Aims:**

We studied the causal association between BMI and lung cancer incidence using observational and MR approaches.

**Methods:**

We followed up 62,453 cancer-free Norwegian adults from 1995–97 (HUNT2) until 2017. BMI at baseline in HUNT2 was classified as < 25.0, 25.0–29.9 and ≥ 30.0 kg/m^2^. BMI change over ten years between HUNT1 (1984–86) and HUNT2 was calculated and classified into quartiles. Seventy-five genetic variants were included as instruments for BMI (among which 14 also associated with smoking behavior). Incident lung cancer cases were ascertained from the Cancer Registry of Norway. Cox regression models were used to estimate hazard ratios (HRs) with 95% confidence intervals (CIs). Multivariable MR was used to examine the effect of BMI after genetically controlling for smoking.

**Results:**

During a median follow-up of 21.1 years, 1009 participants developed lung cancer including 327 with lung adenocarcinoma. The HRs and 95% CIs for incidence of adenocarcinoma were 0.73 (0.58–0.92) for BMI 25.0–29.9 kg/m^2^ and 0.53 (0.37–0.76) for BMI ≥ 30 kg/m^2^ compared with BMI < 25.0 kg/m^2^ in HUNT2 (*P* for trend < 0.001). However, there was little evidence of a dose–response relationship between the BMI change from HUNT1 to HUNT2 in quartiles and the incidence of adenocarcinoma (P for trend = 0.08). Furthermore, multivariable MR approach suggested a positive association between genetically determined 1 kg/m^2^ increase in BMI and the incidence of adenocarcinoma (HR 1.25, 95% CI 1.02–1.53). No associations were found with other lung cancer histologic types.

**Conclusions:**

Our study suggests that the inverse association between baseline BMI and lung adenocarcinoma in observational analysis may not be causal. More MR studies are needed to confirm our finding of a positive association between BMI and lung adenocarcinoma.

**Supplementary Information:**

The online version contains supplementary material available at 10.1186/s12885-022-10215-0.

## Background

Body mass index (BMI) is inversely associated with incidence of lung cancer especially with adenocarcinoma as a major histologic type in previous traditional observational studies [1, 2]. Given that tobacco smoking accounts for around 80–90% of the risk of lung cancer [3] and that there is a complex interrelationship between smoking and BMI [4], residual confounding by smoking may explain for this inverse association [5, 6]. In addition, reverse causation due to weight loss before the lung cancer diagnosis [2] and competing risk due to death associated with obesity [7] may also contribute to the observed inverse association.

On the other hand, the measurements of BMI in most observational studies are attained at one time point [1, 2, 8, 9]. Although BMI change between at least two time points can better examine the potential causal association, BMI change in relation to the incidence of lung cancer has not been well investigated. One case–control study suggested a dose–response inverse association between BMI gain in adulthood and risk of lung cancer, but more evidence from prospective cohort studies is required [10].

Mendelian randomization (MR) approach uses genetic variants as instrumental variables for the risk factor of interest. The advantage of MR is that genetic variants are randomly assigned at conception [11]. Bias due to reverse causation can be avoided and the influence of residual confounding is reduced [11]. Furthermore, genetically determined BMI reflects the level of BMI across the lifespan and therefore is more accurate than a single measurement [12].

However, pleiotropy via smoking may lead to bias in the univariable MR since some of the genetic variants for BMI have been suggested to link with smoking [13]. Multivariable MR, as an extension of univariable MR, can estimate the causal effects of multiple risk factors simultaneously [14, 15]. To date, there is only one such study on this topic showing an inverse association between BMI and incidence of adenocarcinoma but a positive association of BMI with small cell lung cancer [13]. The finding of the inverse association with lung adenocarcinoma in this multivariable MR study is inconsistent with results from the previous univariable MR studies which demonstrated no association [12, 16, 17]. Thus, more multivariable MR studies are needed to clarify the potential associations and thereby to improve our understanding on the complex biological mechanisms underlying different lung cancer histologic types.

To study the causal relationships between BMI and incidence of lung cancer overall and histologic types, we first applied observational analyses using both BMI and BMI change as exposures in a large homogenous population of Norwegian adults who were followed up for over 20 years. Secondly, the possible causal associations were examined using a one-sample multivariable MR approach genetically controlling for smoking.

## Methods

### Study design and population

We used data from the second survey of The Trøndelag Health Study (HUNT2, 1995–97) as the baseline. In total, 65,227 adults (69% of the invited) participated in HUNT2 [18] and were followed up until the date of first diagnosis of lung cancer, the date of death or emigration from Norway or the end of follow-up on December 31, 2017, whichever came first. Lung cancer diagnoses across histologic types were obtained from the Cancer Registry of Norway. Information on vital status and emigration was obtained from the National Population Registry.

**Fig. 1:**
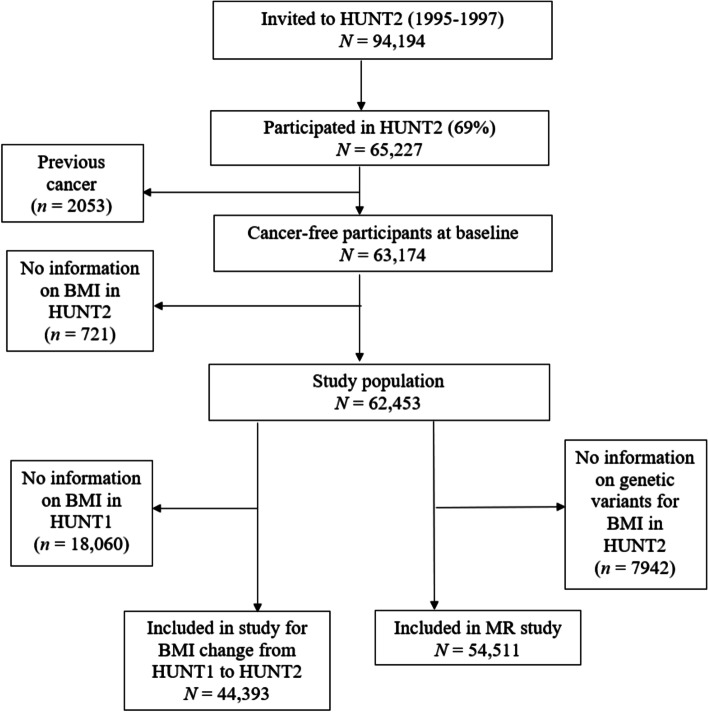
Flow chart of study participants

We first excluded 2053 participants with previous cancer diagnoses before the baseline based on information from the Cancer Registry of Norway (Fig. 1). Afterwards 721 participants with missing information on BMI in HUNT2 were excluded, leaving 62,453 adults for the analysis between BMI in HUNT2 and lung cancer incidence. We additionally excluded 18,060 participants with missing information on BMI from the first survey of The Trøndelag Health Study (HUNT1, 1984–1986), leaving 44,393 participants for the analysis between BMI change from HUNT1 to HUNT2 and lung cancer incidence. Among the 18,060 participants who had missing information on BMI in HUNT1, about 50% of them were not eligible to participate in HUNT1 due to age < 20 years. In the multivariable MR analysis, 7942 participants without information on genetic variants for BMI in HUNT2 were excluded and 54,511 participants were included (Fig. 1).

### BMI, BMI change and genetic variants for BMI

In both HUNT1 and HUNT2, weight and height were measured by health professionals at clinical examination. Height was measured to the nearest centimeters and weight to the nearest 0.5 kg. BMI was calculated as weight in kilograms divided by height squared in meter (kg/m^2^). BMI in HUNT2 was initially categorized into < 18.5, 18.5–24.9, 25.0–29.9 30.0–34.9 and ≥ 35.0 kg/m^2^ according to the recommendations of the World Health Organization (WHO) [19]. Due to limited lung cancer cases in the BMI categories < 18.5 (*n* = 11) and ≥ 35.0 kg/m^2^ (*n* = 30), the BMI categories were collapsed into three groups such as: < 25.0, 25.0–29.9 and ≥ 30.0 kg/m^2^. BMI change from HUNT1 to HUNT2 was categorized into quartiles [1^st^ (-21.3–0.5), 2^nd^ (0.6–1.7), 3^rd^ (1.8–3.1) and 4^th^ (3.2–18.6) kg/m^2^].

DNA samples were extracted from blood samples that were collected in HUNT2 and stored in the HUNT Biobank. Genome-wide genotyping and imputation were carried out for all participants in HUNT2 with sample and variant quality control by using Illumina Humina HumanCoreExome arrays [20]. Seventy-seven single nucleotide polymorphisms (SNPs) were suggested as candidate instrumental variables for BMI with a *P* value < 5 × 10^–8^ based on European sex-combined analyses in a genome-wide association study of the Genetic Investigation of Anthropometric Traits consortium (GIANT) [21]. Information on 2 SNPs (rs12016871 and rs2033732) was missing in the HUNT data since they did not pass imputation quality control, leaving 75 BMI-associated SNPs for our analysis. We then split the remaining 75 BMI-associated SNPs into two groups [13, 15]: 1) 61 SNPs that only affected BMI (BMI-only SNPs) and 2) 14 SNPs that affected both BMI and smoking (BMI & smoking SNPs). The 14 BMI & smoking SNPs were identified based on a P value < 0.05 for the association between each of the 75 BMI-associated SNPs and smoking status in our study population.

### Other baseline variables

Regarding smoking, participants were classified into never, former [≤ 10.0, 10.1–20.0 and > 20.1 pack-years (pyrs)] and current (≤ 10.0, 10.1–20.0 and > 20.1 pyrs). Other covariates were categorized as: sex (women and men), passive smoking (never, only childhood, only adulthood, and both), alcohol consumption (never, 1–4, and ≥ 5 times/month), leisure physical activity (inactive and active), total sitting time daily (0–4, 5–7, and ≥ 8 h), occupational activity (most sedentary work, much walking or lifting at work, and heavy physical work), education (< 10 and ≥ 10 years), economic difficulty (yes and no), family history of cancer (yes and no), self-reported chronic obstructive pulmonary disease (COPD) (yes and no) and asthma (no asthma, non-active asthma, and active asthma). Missing information on each of the aforementioned variables was included in the analyses as an “unknown” category.

### Ascertainment of lung cancer

By using the unique 11-digit personal identification number, participants’ information from HUNT2 was linked to the Cancer Registry of Norway [22]. The International Classification of Diseases version 10 (ICD-10) codes used for registration of lung cancer are C33-C34 [22]. Histologic types were classified according to International Classification of Diseases of Oncology (ICD-O) [23]. Data from the Cancer Registry of Norway are reported to be reasonably accurate and complete [24].

### Statistical analysis

Baseline characteristics of the participants were presented by BMI categories in HUNT2. Cox proportional hazard models were used to examine the associations with incidence of lung cancer. We assessed the proportional hazards assumption by Schoenfeld residuals for exposures and all covariates. *Tvc* option of the *stcox* command in Stata was used if hazards were non-proportional. Crude and adjusted hazard ratios (HRs) with 95% confidence intervals (CIs) were calculated with age as the underlying time variable. Potential confounders were selected based on previous knowledge [25–27]. Included confounders in the main model were sex, smoking status combined with pack-years, passive smoking, physical activity, total sitting time daily, education, economic difficulties, family history of cancer, and self-reported COPD. Asthma, alcohol consumption, and occupational activity were adjusted in an additional model.

We performed several sensitivity analyses to test the robustness of our observational findings: 1) To address reverse causality due to existing but undiagnosed lung cancer, we excluded the first five years of follow-up. 2) Potential competing risk due to death was investigated using the Fine-Gray model [28]. 3) Residual confounding due to information missing was investigated by multivariable chained imputation with fully conditional specification (m = 10 imputed datasets) for all covariates based on the assumption of missing at random. 4) To further address residual confounding by smoking, we performed negative control exposure analysis by using migraine as an alternative exposure [29] (details described in Supplementary Text 1). Previous study suggested that migraine was associated with smoking [30, 31] but not with lung cancer.

Furthermore, we performed a multivariable MR study to assess the potential causal association between genetically predicted BMI and incidence of lung cancer genetically controlling for the influence of smoking status. All 75 BMI-associated SNPs (61 BMI-only SNPs and 14 BMI & smoking SNPs) were used as instrument variables and r^2^ measure of linkage disequilibrium among instruments were < 0.01 at a 10-MB window [21]. BMI and smoking status in HUNT2 were both regarded as exposures in the multivariable MR. We obtained estimates on SNPs–BMI, SNPs–smoking (regarded as an ordinal variable) and SNPs–lung cancer associations from the same individuals and applied two-sample MR methods such as the inverse variance weighted (IVW) and MR-Egger methods. Both methods could be used in a one-sample setting [32]. We calculated coefficients and standard errors with adjustment for sex, age and age-squared for the SNPs-BMI associations [21]. No adjustments were made for the SNPs-smoking and SNPs-lung cancer associations since no associations between the 75 SNPs and the other confounders were found except for smoking. Sanderson-Windmeijer conditional F-statistics was used to estimate the strength of the instruments for BMI conditional on smoking [33]. Cochran’s Q tests for both IVW and MR-Egger were used to detect heterogeneity between the ratio estimates of SNPs. The intercept test of the MR-Egger was used to assess possibility of horizontal pleiotropy. The outliers in both multivariable IVW and MR-Egger regression methods were identifed using the MR-Pleiotropy Residual Sum and Outlier (MR-PRESSO) [34] method.

We also applied different univariable MR methods based on the 61 BMI-only SNPs as sensitivity analyses to test the robustness of the results from the multivariable MR analysis: 1) Two-stage method based on the weighted BMI genetic risk score (details described in Supplementary Text 2). 2) IVW and MR-Egger methods using summary data of the 61 individual genetic variants (Supplementary Text 2). All statistical analyses were performed with STATA/SE 16.1 (College Station, TX, USA) or R (4.0.2). The package “MendelianRandomization” was used for the multivariable MR in R.

## Results

In total, 1009 out of the 62,453 participants developed lung cancer during a median follow-up of 21.1 years, among which 327 were adenocarcinoma. Compared to those with BMI < 25.0 kg/m^2^, participants with BMI 25.0–29.9 and ≥ 30.0 kg/m^2^ were older, more likely to be former smokers or non-drinkers, less active or lower educated and were more likely to have a family history of cancer at baseline (Table 1).

**Table 1:** Baseline characteristics according to BMI categories in the HUNT2 Study, 1995–1997 (*N* = 62,453)

	**BMI (kg/m**^**2**^**)**		
**Variables**	** < 25.0**	**25.0–29.9**	** ≥ 30.0**
Number of subjects	25,056	27,115	10,282
Age (years)	45.5 ± 17.0	51.4 ± 16.4	54.1 ± 16.3
Number of lung cancer cases (%)	435 (43.1)	426 (42.2)	148 (14.7)
Sex, % (women/men)	58.8/41.2	45.2/54.8	58.7/41.3
Smoking, % (never/current/former/unknown)	42.3/34.8/21.0/2.0	42.0/25.8/30.3/2.0	44.4/22.2/30.9/2.5
Passive smoking, % (never/ever/unknown)	19.2/79.2/1.5	17.8/80.3/1.9	16.8/80.9/2.3
Alcohol consumption (times/month), % (never/ ≥ 1/unknown)	30.8/61.4/7.9	33.7/57.5/8.9	45.2/45.2/9.6
Leisure physical activity, % (inactive^a^/active^b^/unknown)	19.7/53.2/27.1	21.3/47.6/31.2	26.6/37.1/36.3
Total sitting time daily (hours), % (< 8/ ≥ 8/unknown)	49.1/28.4/22.5	49.2/28.2/22.6	46.8/27.3/25.9
Occupational activity (Most sedentary/much walking or lifting/heavy physical work/unknown)	23.1/46.9/8.9/21.1	23.1/41.1/10.9/24.9	21.4/36.7/9.0/32.9
Education (years), % (< 10/ ≥ 10/unknown)	27.7/68.3/4.0	35.9/59.1/5.0	44.8/47.7/7.5
Economic difficulties, % (no/yes/unknown)	50.4/22.5/27.1	48.9/20.4/30.7	41.8/23.3/34.9
Family history of cancer, % (no/yes)	77.4/22.6	73.1/27.0	71.9/28.1
COPD^c^, % (no/yes)	98.1/1.9	97.8/2.2	96.9/3.1
Asthma, % (no/non–active/active/unknown)	95.0/1.8/3.2/0.1	94.7/1.7/3.6/0.1	92.6/2.0/5.4/0.1

Data are given as mean ± standard deviation or percentage of subjects in each BMI category

Abbreviations: *BMI* body mass index, *COPD* chronic obstructive pulmonary disease, *HUNT* The Trøndelag Health Study

^a^Inactive: no physical activity or only light physical activity ≤ 2 h per week

^b^Active: physical activity level from low to high

^c^COPD: self-reported COPD

In the observational analyses, BMI in HUNT2 was inversely associated with the incidence of lung cancer overall after adjustment for smoking and other confounders (Table 2). The HR was 0.79 (95% CI 0.69–0.91) for BMI 25.0–29.9 kg/m^2^ and 0.75 (95% CI 0.62–0.91) for BMI ≥ 30.0 kg/m^2^ compared with BMI < 25.0 kg/m^2^. There was a stronger inverse association between BMI and lung adenocarcinoma (*P* for trend < 0.001), and the corresponding HRs were 0.73 (95% CI 0.58–0.92) and 0.53 (95% CI 0.37–0.76). The corresponding HRs for 1 kg/ m^2^ increase in BMI was 0.95 (95% CI 0.92–0.98) for adenocarcinoma. No clear association was found for incidence of small cell or squamous cell lung cancer (Table 2). Additional adjustment for asthma, alcohol consumption, and occupational activity did not change the results markedly.

**Table 2:** The association of BMI in HUNT2 with incidence of lung cancer overall and different histologic types, the HUNT Study, 1995–97 to 2017 (*N* = 62,453)

LC	BMI (kg/m^2^)	Cases	IR (per 1000 person-years)	Crude model^a^		Main model^b^		Additional Model^c^		Main Model after imputation^d^	
				**HR**	**95% CI**	**HR**	**95% CI**	**HR**	**95% CI**	**HR**	**95% CI**
Overall	< 25.0	435	0.90	1.00	Reference	1.00	Reference	1.00	Reference	1.00	Reference
	25.0–29.9	426	0.84	0.65	0.57–0.74	0.79	0.69–0.91	0.79	0.69–0.91	0.80	0.70–0.92
	≥ 30.0	148	0.80	0.54	0.45–0.66	0.75	0.62–0.91	0.75	0.62–0.91	0.77	0.64–0.93
SC	< 25.0	62	0.13	1.00	Reference	1.00	Reference	1.00	Reference	1.00	Reference
	25.0–29.9	66	0.13	0.71	0.50–1.00	0.97	0.68–1.38	0.96	0.68–1.36	0.98	0.69–1.39
	≥ 30.0	29	0.16	0.75	0.48–1.17	1.13	0.72–1.77	1.11	0.71–1.74	1.15	0.73–1.80
AD	< 25.0	150	0.31	1.00	Reference	1.00	Reference	1.00	Reference	1.00	Reference
	25.0–29.9	139	0.27	0.63	0.50–0.79	0.73	0.58–0.92	0.73	0.58–0.92	0.73	0.58–0.93
	≥ 30.0	38	0.20	0.42	0.29–0.60	0.53	0.37–0.76	0.53	0.37–0.76	0.53	0.37–0.76
SQ	< 25.0	83	0.17	1.00	Reference	1.00	Reference	1.00	Reference	1.00	Reference
	25.0–29.9	86	0.17	0.67	0.50–0.91	0.81	0.60–1.10	0.81	0.60–1.11	0.82	0.61–1.12
	≥ 30.0	30	0.16	0.56	0.37–0.85	0.84	0.55–1.28	0.83	0.54–1.27	0.88	0.57–1.34

*Tvc* option of the stcox command in Stata was used to model the non-proportional hazards in the main, additional, and imputed models. Non-proportional hazards for LC overall: sex, smoking and economic difficulties; for SC: smoking, family history of cancer, economic difficulties, and leisure physical activity; for AD: sex, smoking, economic difficulties and leisure physical activity; for SQ: education

Abbreviations: *AD* adenocarcinoma, *CI* confidence interval, *HR* hazard ratio, *HUNT* The Trøndelag Health Study, *IR* incidence rate, *LC* lung cancer, *SC* small cell lung cancer, *SQ* squamous cell lung cancer

^a^Age was used as the time scale in the crude model

^b^Main model adjusted for sex, smoking, passive smoking, leisure physical activity, total sitting time daily, education, economic difficulties, family history of cancer, and self-reported chronic obstructive pulmonary disease (COPD). Age was used as the time scale

^c^Adjusted for covariables in the main model plus asthma, alcohol consumption, and occupational activity. Age was used as the time scale

^d^Missing information in the covariates of the main model were imputed

Results of the sensitivity analyses provided supportive evidence for the above findings. 1) After excluding the first five years of follow-up, the association estimate of BMI in HUNT2 was slightly attenuated for lung cancer overall (*n* = 854) but remained similar for adenocarcinoma (*n* = 281) (Supplementary Table S1). 2) The competing risk analysis showed similar results to our primary results even if there were many cases of death (*n* = 15,472) (Supplementary Table S2). 3) Multiple imputation for all covariates including smoking showed comparable association estimates between BMI in HUNT2 and adenocarcinoma. 4) In the analysis of using migraine as a negative control exposure (Supplementary Text 1, Supplementary Fig. 1, and Supplementary Table S3), no clear association between migraine and incidence of adenocarcinoma was demonstrated, suggesting that our observed inverse association of BMI in HUNT2 with adenocarcinoma was less likely to be explained by residual confounding from smoking.

Despite an inverse linear trend between BMI measured at one time point in HUNT2 and adenocarcinoma, there was little evidence of a dose–response relationship between the BMI change from HUNT1 to HUNT2 in quartiles and the incidence of adenocarcinoma (P for trend = 0.08, Table 3). Compared to participants with BMI change in the 1^st^ quartile (-21.3–0.5), participants with BMI change of 0.6–1.7, 1.8–3.1 and 3.2–18.6 kg/m^2^ had a HR of 0.68 (95% CI 0.49–0.92), 0.68 (95% CI 0.49–0.94), and 0.78 (95% CI 0.56–1.09), respectively. Compared with participants who had information on BMI change from HUNT1 to HUNT2 (*n* = 44,393), those without information on the BMI change (*n* = 18,060) were younger, more physically active and had higher socio-economic status (data not presented).

**Table 3:** The association of BMI change from HUNT1 to HUNT2 with incidence of lung cancer overall and different histologic types, the HUNT Study (*N* = 44,393)

LC	BMI change in quartiles (kg/m^2^)	Cases	IR (per 1000 person-years)	Crude model^a^		Main model^b^		Additional Model^c^		Main Model after imputation^d^	
				**HR**	**95% CI**	**HR**	**95% CI**	**HR**	**95% CI**	**HR**	**95% CI**
Overall	1st (-21.3–0.5)	363	1.96		Reference	1.00	Reference	1.00	Reference	1.00	Reference
	2nd (0.6–1.7)	215	1.08	0.63	0.53–0.74	0.78	0.66–0.93	0.78	0.66–0.93	0.80	0.67–0.95
	3rd (1.8–3.1)	177	0.81	0.52	0.43–0.62	0.71	0.59–0.85	0.71	0.59–0.86	0.73	0.61–0.88
	4th (3.2–18.6)	153	0.75	0.54	0.44–0.65	0.75	0.62–0.91	0.75	0.61–0.91	0.78	0.64–0.95
SC	1st (-21.3–0.5)	62	0.33	1.00	Reference	1.00	Reference	1.00	Reference	1.00	Reference
	2nd (0.6–1.7)	20	0.10	0.33	0.20–0.55	0.44	0.26–0.73	0.45	0.27–0.74	0.45	0.27–0.75
	3rd (1.8–3.1)	23	0.11	0.38	0.23–0.62	0.54	0.33–0.88	0.55	0.34–0.90	0.57	0.35–0.92
	4th (3.2–18.6)	27	0.13	0.53	0.34–0.84	0.74	0.46–1.19	0.75	0.47–1.21	0.80	0.49–1.28
AD	1st (-21.3–0.5)	112	0.60	1.00	Reference	1.00	Reference	1.00	Reference	1.00	Reference
	2nd (0.6–1.7)	63	0.32	0.57	0.42–0.78	0.68	0.49–0.92	0.67	0.49–0.91	0.69	0.50–0.94
	3rd (1.8–3.1)	61	0.28	0.55	0.40–0.75	0.68	0.49–0.94	0.67	0.49–0.92	0.69	0.50–0.95
	4th (3.2–18.6)	60	0.29	0.63	0.46–0.87	0.78	0.56–1.09	0.77	0.55–1.07	0.80	0.58–1.12
SQ	1st (-21.3–0.5)	62	0.33	1.00	Reference	1.00	Reference	1.00	Reference	1.00	Reference
	2nd (0.6–1.7)	53	0.27	0.92	0.63–1.32	1.18	0.82–1.71	1.19	0.82–1.73	1.21	0.84–1.76
	3rd (1.8–3.1)	47	0.22	0.82	0.56–1.21	1.24	0.84–1.83	1.26	0.85–1.85	1.29	0.88–1.91
	4th (3.2–18.6)	21	0.10	0.44	0.27–0.73	0.77	0.46–1.28	0.78	0.47–1.30	0.82	0.49–1.37

*Tvc* option of the stcox command in Stata was used to model the non-proportional hazards in the main, additional, and imputed models. Non-proportional hazards for LC overall: sex, smoking, and economic difficulties; for SC: smoking; for AD: sex, smoking, economic difficulties; for SQ: education

Abbreviations: *AD* adenocarcinoma, *BMI* body mass index, *CI* confidence interval, *HR* hazard ratio, *HUNT* The Trøndelag Health Study, *IR* incidence rate, *LC* lung cancer, *SC* small cell lung cancer, *SQ* squamous cell lung cancer

^a^Age was used as the time scale in the crude model

^b^Main model adjusted for sex, smoking, passive smoking, leisure physical activity, total sitting time daily, education, economic difficulties, family history of cancer, and self-reported chronic obstructive pulmonary disease (COPD). Age was used as the time scale

^c^Adjusted for covariables in the main model plus asthma, alcohol consumption, and occupational activity. Age was used as the time scale

^d^Missing information in the covariates of the main model were imputed

The MR analyses were performed in 54,511 of the 62,453 participants with available BMI-associated SNPs. There were no major differences in most of the baseline characteristics between participants with (*n* = 54,511) and without (*n* = 7942) information on the SNPs, except for alcohol consumption and physical activity level showing relatively larger difference in the two populations (Supplementary Table S4). The F statistic value of the genetic risk score including 75 BMI-associated SNPs was 1174 and it explained 2.0% of the variance of BMI in HUNT2. The conditional F statistic was 242, which suggested that the SNPs used in the multivariable MR were good instruments for BMI conditional on smoking. Results from the Cochran’s Q tests for adenocarcinoma suggested possible heterogeneity of the ratio estimates (*P* for Q = 0.01, Table 4). After removing one outlier SNP (rs2121279) identified by the MR-PRESSO method, there was no heterogeneity between the remaining ratio estimates (*P* for Q = 0.09, Table 4). Each 1 kg/m^2^ increment in genetically determined BMI increased the incidence of adenocarcinoma by 25% (HR 1.25, 95% CI 1.02–1.53) in the multivariable MR analysis after removal of the outlier (Table 4). Multivariable MR-Egger method showed similar result (HR 1.52, 95% CI 1.09–2.11). There was no evidence of association between genetically determined BMI and incidence of other histologic types. The intercept test from MR-Egger suggested the pleiotropy was balanced as the P value for the intercept test was > 0.05. Finally, results from the univariable MR analyses using the 61 BMI-only SNPs showed similar results as in the multivariable MR analysis with wider 95% CIs (Supplementary Text 2, Supplementary Table S5 and Supplementary Table S6).

**Table 4:** The association of BMI with incidence of lung cancer overall and different histologic types based on the multivariable MR analysis, the HUNT Study, 1995–97 to 2017 (*N* = 54,511)

**LC**	**Cases**		**Multivariable MR-IVW**			**Multivariable MR-Egger**			
			**HR**^**1**^	**95% CI**	**P for Q**^**2**^	**HR**^**1**^	**95% CI**	**P for Q**^**2**^	**P for inter**^**3**^
Overall	873		1.07	0.96 –1.19	0.51	1.16	0.98–1.38	0.55	0.23
SC	136		1.08	0.83–1.42	0.79	1.32	0.85–2.04	0.81	0.26
AD	289		1.28	1.03–1.58	0.01	1.45	1.01–2.06	0.01	0.39
		Outlier-corrected^4^	1.25	1.02–1.53	0.09	1.52	1.09–2.11	0.11	0.14
SQ	177		0.96	0.76–1.21	0.99	0.89	0.60–1.31	0.99	0.66

Abbreviations: *AD* adenocarcinoma, *BMI* body mass index, *CI* confidence interval, *HR* hazard ratio, *HUNT* The Trøndelag Health Study, *IVW* inverse variance weighted method, *LC* lung cancer, *MR* Mendelian randomization, *SC* small-cell lung cancer, *SQ* squamous cell lung cancer

^1^Per 1 unit (kg/m^2^) increase in genetically determined BMI

^2^*P* value for Cochran’s Q test

^3^*P* value for intercept test of multivariable MR-Egger regression

^4^Results after excluding the outlier SNP (rs2121279) for adenocarcinoma

## Discussion

In the observational analyses, we found BMI at baseline was inversely associated with the incidence of lung adenocarcinoma in a dose–response fashion. However, the dose–response relationship was not supported by the results of the BMI change. In the multivariable MR analysis, we observed a positive association between genetically determined BMI and the incidence of adenocarcinoma after taking smoking into account. There was no evidence of associations with other histologic types in either the observational or the MR analysis.

Our observed inverse association between BMI and incidence of adenocarcinoma was consistent with previous observational studies [1, 2]. However, many previous studies might suffer from insufficient adjustment for smoking, reverse causation due to preclinical weight loss and/or competing risk of death. We performed several sensitivity analyses such as multiple imputation and negative control exposure by migraine to address the possibility of residual confounding by smoking. We also excluded the first five years of follow-up to address the possibility of reverse causation and performed analysis to address competing risk of death. Results from these sensitivity analyses appeared to support the inverse association between BMI at baseline and adenocarcinoma.

However, this observed inverse association may not be interpreted as a causal association for the following reasons: 1) Unlike the results of BMI at baseline, our results of BMI change over ten years in the adulthood did not show a dose–response relationship with the incidence of adenocarcinoma. 2) We were not able to completely exclude the residual confounding by smoking although we had attempted to address it in several ways. This is supported by the findings from two large cohort studies of 1.2 million women in the UK [5, 6], in which no clear association between BMI and incidence of lung cancer overall was found in never smokers. 3) Unmeasured or unknown confounders always exist in observational studies. Obesity is accompanied with many other lifestyle factors, some of which may not be measured or are unknown. 4) Our multivariable MR analysis suggested a positive association between BMI and the incidence of adenocarcinoma instead of an inverse association.

Using genetic variants as proxies of BMI, MR studies avoid reverse causation and have a better control for residual confounding [11]. Additionally, in contrast to observational studies, MR studies can reduce bias due to measurement errors of BMI and reflect the BMI level across the lifespan. To date, there have been limited MR studies and the findings are inconsistent [12, 13, 16, 17]. Most of these studies have applied an univariable MR approach [12, 16, 17]. The interaction between BMI and smoking in the development of lung cancer seems complicated [13, 16]. Thus, the assumption of no horizontal pleiotropy may be violated in the univariable MR without taking account of smoking. By using multivariable MR approach, however, the effects of BMI and smoking on lung cancer can be jointly examined [13]. Unlike our finding, Zhou et al. found an inverse association between BMI and risk of adenocarcinoma after controlling for smoking in a two-sample multivariable MR [13]. However, the study by Zhou et al. might be subjected to weak instruments bias since the value of conditional F statistics was below 10 [13]. In contrast, our result of conditional F statistics was 242 and thereby the genetic variants in our study seemed to be better instruments for BMI after controlling for smoking. In addition, it was not possible to check the assumption of independence in the study by Zhou et al. using a two-sample MR [13]. In our one-sample MR study, we had the possibility to thoroughly check for the associations between BMI-associated SNPs and the important confounders and further adjusted for self-reported COPD when calculating the effect estimates between the BMI-only SNPs and adenocarcinoma. The effect estimates showed similar trends in our multivariable MR using all the BMI-associated SNPs and in the univariable MR using the BMI-only SNPs.

The mechanisms for a positive association between BMI and lung adenocarcinoma are unknown but some possibilities have been suggested: 1) High insulin resistance related to obesity might contribute to the lung carcinogenesis [35, 36]. 2) Adipokines (leptin and adiponectin) secreted by the adipose tissue might affect the progress of carcinogenesis through irregular immunomodulation or chronic inflammation [36, 37]. Although we did not find associations, previous MR studies reported positive associations between BMI and small cell or squamous cell lung cancer [12, 16]. Thus, the impact of BMI on the incidence of lung cancer histologic types and the underlying mechanisms warrants further investigation. The latest lung cancer genome-wide association studies identified distinct genetic variants for different lung histologic types [38, 39]. For example, the identified variants specifically for lung adenocarcinoma are near genes related to lung function, telomere regulation and endogenous DNA damage. Therefore, different mechanisms may exist for different histologic types. If positive associations were confirmed in future studies, control of body weight could be another preventive measure to reduce the incidence of lung cancer in addition to smoking cessation.

Our study is the first study that has explored the association between BMI and the incidence of lung cancer using different approaches in a large and homogenous population. A large and homogeneous study population can minimize population stratification bias [40]. A long follow-up duration over 20 years made it possible to include a large number of lung cancer cases and the statistical power was ample. We also had objective measurements of BMI and validated information of lung cancer cases from the Cancer Registry of Norway [22] which reduced bias due to misclassification. The F statistic values including the conditional F statistic suggested that the BMI-associated SNPs were good instruments.

However, our study has several limitations. Firstly, selection bias cannot be completely ruled out for the MR results since 13% (7942/62453) of the participants had no genetic information on BMI. The excluded participants in the MR analyses had lower alcohol consumption and lower physical activity level than the included participants. However, the proportion of BMI ≥ 25.0 kg/m^2^ (60.0% & 59.9%) and the proportion of lung cancer cases (1.7% & 1.6%) were similar between the excluded and included participants. Secondly, we could not exclude the possibility of pleiotropy for the association between BMI and adenocarcinoma since there was evidence of heterogeneity in BMI-associated SNPs in both the multivariable and univariable MR analyses before the outlier was removed. Nevertheless, our results from the multivariable MR-Egger, which was relatively robust for horizontal pleiotropy, suggested the pleiotropic effects were balanced around the overall effect. After removing the outlier, no heterogeneity was shown.

## Conclusion

Overall, our study suggests that the inverse association between BMI and the incidence of lung adenocarcinoma in the observational analyses may not be causal. Reverse causation and residual confounding by smoking were less likely to explain for the observed inverse association. More multivariable MR studies using large individual data are needed to confirm or refute our finding of a positive association between BMI and lung adenocarcinoma.

## Supplementary Information

**Additional file 1: Supplementary Table S1.** The association of BMI in HUNT2 with incidence of lung cancer overall and different histologic types after excluding the first five years’ follow-up, the HUNT Study, 1995-97 to2017 (*N* = 59,711). **Supplementary Table S2. **The associations of BMI in HUNT2 with lung cancer incidence taking account of competing risk due to death, the HUNT Study, 1995-97 to 2017 (*N* = 62,453). **Supplementary Text 1.** Analysis using negative control exposure. **Supplementary Figure 1.** DAG for body mass index(BMI) (as the main exposure), migraine (as the negative control exposure) and incidence of lung cancer (as the outcome). **Supplementary Table S3.** Negative control using migraine as an alternative exposure to address residual confounding by smoking for the association of BMI in HUNT2 with lung cancer incidence across different lung cancer subtypes, the HUNT Study, 1995-97 to 2017(*N* = 49,969). **Supplementary Table S4.** Comparison of baseline characteristics of participants with complete and missing information on 75 BMI SNPs in HUNT2. **Supplementary Text 2.** Univariable Mendelian randomization (MR) analyses using the 61 single-nucleotide polymorphisms only for body mass index (BMI-Only SNPs). **Supplementary Table S5. **Associations of externally weighted BMI GRS^1^ based on 61 BMI-Only SNPs with potential confounders in HUNT2, 1995-1997 (*N* = 54,511). **Supplementary Table S6. **The association of BMI with incidence of lung cancer overall and different histologic types based on the univariable MR analyses using 61 BMI-Only SNPs,the HUNT Study, 1995-97 to 2017 (*N* = 54,511).

## Data Availability

Data from the HUNT Study is available on request to the HUNT Data Access Committee (hunt@ medisin.ntnu.no) when is used in research projects. The HUNT data access information describes the policy regarding data availability (https://www.ntnu.edu/hunt/data).

## Abbreviations

BMI: Body mass index
CI: Confidence interval
COPD: Chronic obstructive pulmonary disease
GIANT: Genetic Investigation of Anthropometric Traits consortium
HR: Hazard ratio
HUNT: The Trøndelag Health Study
ICD-10: International Classification of Diseases version 10
ICD-O: International Classification of Diseases of Oncology
IVW: Inverse variance weighted
MR: Mendelian randomization
MR-PRESSO: MR- Pleiotropy Residual Sum and Outlier
SNPs: Seventy-seven single nucleotide polymorphisms
WHO: World Health Organization
