# Supplementary material for: Body mass index and incidence of lung cancer in the HUNT study: using observational and Mendelian randomization approaches
Source: BMC Cancer. 2022 Nov 8;22:1152. doi: 10.1186/s12885-022-10215-0 (PMC9644519; doi:10.1186/s12885-022-10215-0)
Supplement: Supplementary file 1 — Additional file 1: Supplementary Table S1. The association of BMI in HUNT2 with incidence of lung cancer overall and different histologic types after excluding the first five years’ follow-up, the HUNT Study, 1995-97 to2017 (N = 59,711). Supplementary Table S2. The associations of BMI in HUNT2 with lung cancer incidence taking account of competing risk due to death, the HUNT Study, 1995-97 to 2017 (N = 62,453). Supplementary Text 1. Analysis using negative control exposure. Supplementary Figure 1. DAG for body mass index(BMI) (as the main exposure), migraine (as the negative control exposure) and incidence of lung cancer (as the outcome). Supplementary Table S3. Negative control using migraine as an alternative exposure to address residual confounding by smoking for the association of BMI in HUNT2 with lung cancer incidence across different lung cancer subtypes, the HUNT Study, 1995-97 to 2017(N = 49,969). Supplementary Table S4. Comparison of baseline characteristics of participants with complete and missing information on 75 BMI SNPs in HUNT2. Supplementary Text 2. Univariable Mendelian randomization (MR) analyses using the 61 single-nucleotide polymorphisms only for body mass index (BMI-Only SNPs). Supplementary Table S5. Associations of externally weighted BMI GRS1 based on 61 BMI-Only SNPs with potential confounders in HUNT2, 1995-1997 (N = 54,511). Supplementary Table S6. The association of BMI with incidence of lung cancer overall and different histologic types based on the univariable MR analyses using 61 BMI-Only SNPs,the HUNT Study, 1995-97 to 2017 (N = 54,511). [file 12885_2022_10215_MOESM1_ESM.docx]

Supplementary Table S1. The association of BMI in HUNT2 with incidence of lung cancer overall and different histologic types after excluding the first five years’ follow-up, the HUNT Study, 1995-97 to 2017 (N = 59,711)

| LC | BMI (kg/m^2^) | Cases | IR (per 1000 person-years) | Crude model^1^ | | Main model^2^ | | Additional Model^3^ | |
| --- | --- | --- | --- | --- | --- | --- | --- | --- | --- |
|  |  |  |  | **HR** | **95% CI** | **HR** | **95% CI** | **HR** | **95% CI** |
| Overall | <25.0 | 360 | 1.00 | 1.00 | Reference | 1.00 | Reference | 1.00 | Reference |
|  | 25.0-29.9 | 362 | 0.96 | 0.67 | 0.57–0.77 | 0.81 | 0.70–0.94 | 0.81 | 0.70–0.94 |
|  | ≥30.0 | 132 | 0.98 | 0.60 | 0.49–0.73 | 0.81 | 0.66–0.99 | 0.80 | 0.66–0.99 |
|  | Continuous BMI^4^ | 854 |  | 0.94 | 0.92–0.96 | 0.98 | 0.96–0.99 | 0.97 | 0.96–0.99 |
| SC | <25.0 | 55 | 0.15 | 1.00 | Reference | 1.00 | Reference | 1.00 | Reference |
|  | 25.0-29.9 | 59 | 0.16 | 0.72 | 0.50–1.04 | 0.97 | 0.67–1.41 | 0.96 | 0.66–1.39 |
|  | ≥30.0 | 28 | 0.21 | 0.85 | 0.54–1.34 | 1.24 | 0.78–1.97 | 1.20 | 0.75–1.91 |
|  | Continuous BMI^4^ | 142 |  | 0.98 | 0.94–1.02 | 1.02 | 0.98–1.06 | 1.02 | 0.98–1.06 |
| AD | <25.0 | 132 | 0.37 | 1.00 | Reference | 1.00 | Reference | 1.00 | Reference |
|  | 25.0-29.9 | 115 | 0.31 | 0.59 | 0.46–0.76 | 0.69 | 0.53–0.89 | 0.69 | 0.53–0.89 |
|  | ≥30.0 | 34 | 0.25 | 0.44 | 0.30–0.64 | 0.54 | 0.37–0.80 | 0.55 | 0.37–0.80 |
|  | Continuous BMI^4^ | 281 |  | 0.92 | 0.89–0.96 | 0.95 | 0.92–0.98 | 0.95 | 0.92–0.98 |
| SQ | <25.0 | 65 | 0.18 | 1.00 | Reference | 1.00 | Reference | 1.00 | Reference |
|  | 25.0-29.9 | 77 | 0.20 | 0.76 | 0.55–1.06 | 0.90 | 0.64–1.26 | 0.91 | 0.65–1.27 |
|  | ≥30.0 | 26 | 0.19 | 0.63 | 0.40–0.99 | 0.91 | 0.57–1.44 | 0.89 | 0.56–1.42 |
|  | Continuous BMI^4^ | 168 |  | 0.94 | 0.91–0.98 | 0.98 | 0.94–1.02 | 0.98 | 0.94–1.02 |

Abbreviations: AD: adenocarcinoma; CI: confidence interval; HR: hazard ratio; IR: incidence rate; LC: lung cancer; SC: small cell lung cancer; SQ: squamous cell lung cancer.

^1^Age was used as the time scale in the crude model.

^2^ Main model adjusted for sex, smoking, passive smoking, leisure physical activity, total sitting time daily, education, economic difficulties, family history of cancer, and self-reported chronic obstructive pulmonary disease (COPD). Age was used as the time scale.

^3^ Adjusted for covariables in the main model plus asthma, alcohol consumption, and occupational activity. Age was used as the time scale.

*Tvc* option of the *stcox* command in Stata was used to model the non-proportional hazards in the main and additional models. Non-proportional hazards for LC overall: sex, smoking, and economic difficulties; for SC: smoking, family history of cancer, and leisure physical activity; for AD: economic difficulties and leisure physical activity; for SQ: smoking and education.

^4^ Per 1 unit increase in BMI value.

| LC | BMI (kg/m^2^) | n/Cases | IR (per 1000 person-years) | Adjusted^1^ SHR | 95% CI |
| --- | --- | --- | --- | --- | --- |
| Overall | <25.0 | 435 | 0.90 | 1.00 | Reference |
|  | 25.0-29.9 | 426 | 0.84 | 0.81 | 0.71–0.93 |
|  | ≥30.0 | 148 | 0.80 | 0.71 | 0.59–0.86 |
|  | Continuous BMI^2^ | 1009 |  | 0.97 | 0.95–0.98 |
| SC | <25.0 | 62 | 0.13 | 1.00 | Reference |
|  | 25.0-29.9 | 66 | 0.13 | 0.99 | 0.70–1.42 |
|  | ≥30.0 | 29 | 0.16 | 1.08 | 0.68–1.72 |
|  | Continuous BMI^2^ | 157 |  | 1.01 | 0.97–1.05 |
| AD | <25.0 | 150 | 0.31 | 1.00 | Reference |
|  | 25.0-29.9 | 139 | 0.27 | 0.75 | 0.59–0.95 |
|  | ≥30.0 | 38 | 0.20 | 0.50 | 0.35–0.73 |
|  | Continuous BMI^2^ | 327 |  | 0.95 | 0.92–0.98 |
| SQ | <25.0 | 83 | 0.17 | 1.00 | Reference |
|  | 25.0-29.9 | 86 | 0.17 | 0.83 | 0.61–1.13 |
|  | ≥30.0 | 30 | 0.16 | 0.79 | 0.52–1.21 |
|  | Continuous BMI^2^ | 199 |  | 0.97 | 0.93–1.01 |

Supplementary Table S2. The associations of BMI in HUNT2 with lung cancer incidence taking account of competing risk due to death, the HUNT Study, 1995-97 to 2017 (N = 62,453)

Abbreviations: AD: adenocarcinoma; BMI: body mass index; CI: confidence interval; IR: incidence rate; LC: lung cancer; SC: small cell lung cancer; SHR: sub-distribution hazard ratio; SQ: squamous cell lung cancer.

^1^ Adjusted for sex, smoking, passive smoking, physical activity, total sitting time daily, education, economic difficulties, family history of cancer, and self-reported chronic obstructive pulmonary disease (COPD). Age was used as the time scale.

*Tvc* option of the stcox command in Stata was used to model the non-proportional hazards in the adjusted model. Non-proportional hazards for LC overall: sex, smoking and economic difficulties; for SC: smoking, family history of cancer, economic difficulties, and leisure physical activity; for AD: sex, smoking, economic difficulties, and leisure physical activity; for SQ: education.

^2^ Per 1 unit increase in BMI value.

Supplementary Text 1. Analysis using negative control exposure

The aim of analysis using a negative control exposure is to identify residual confounding that may have resulted in invalid causal inference for the main exposure-outcome association ^1^. In the current study, we used “migraine” as the negative control exposure to detect residual confounding by smoking in the observed BMI-lung cancer association (Supplementary Figure). “Migraine” was chosen as the negative control exposuse was because it is associated with the confounder (smoking) ^2,3^, but not causally associated with the outcome (lung cancer). We expected to observe a null association between migraine and lung cancer after adjustment for smoking, which suggested that the observed BMI-lung cancer association was less likely biased by the residual confounding of smoking.

Based on our study population (N = 62,453), we excluded participants without information on headache. This left 49,969 participants to study the relationship between migraine and lung cancer incidence. Participants with migraine were those who answered yes to the question “Have you sufferred from headache during the last 12 months?” and specified the type of headache as “migraine”, and the rest were regarded as no migraine. We adjusted for smoking status in model 1. In model 2, we adjusted for the same confounders as in our primary study (Supplementary Table S).

At baseline in HUNT2, fewer participants with migraine were heavy smokers (> 20.1 pack-years) than participants without migraine (6.4% vs 10.5%). There was an inverse association between migraine and lung cancer overall incidence without adjustment for smoking (crude HR 0.68, 95% CI 0.49 to 0.93). After adjustment for smoking the association between migraine and lung cancer overall became less clear (HR 0.83, 95% CI 0.60 to 1.14), and additional adjustment for the same confounders as in the primary study did not have material changes in the results. Similarly, migraine was associated with an imprecisely reduced HR for adenocarcinoma before adjustment for smoking (HR 0.85, 95 CI 0.52 to 1.39). The HR became much weaker after adjustment for smoking (HR 0.99, 95% 0.61 to 1.63).

In summary, we didn’t observe a clear association of migraine with incidence of lung cancer overall or adenocarcinoma after adjustment for smoking. This suggested that our observed inverse associations of BMI in HUNT2 with lung cancer incidence overall and adenocarcinoma were less likely to be biased by residual confounding due to smoking.

Supplementary Figure 1. DAG for body mass index (BMI) (as the main exposure), migraine (as the negative control exposure) and incidence of lung cancer (as the outcome)


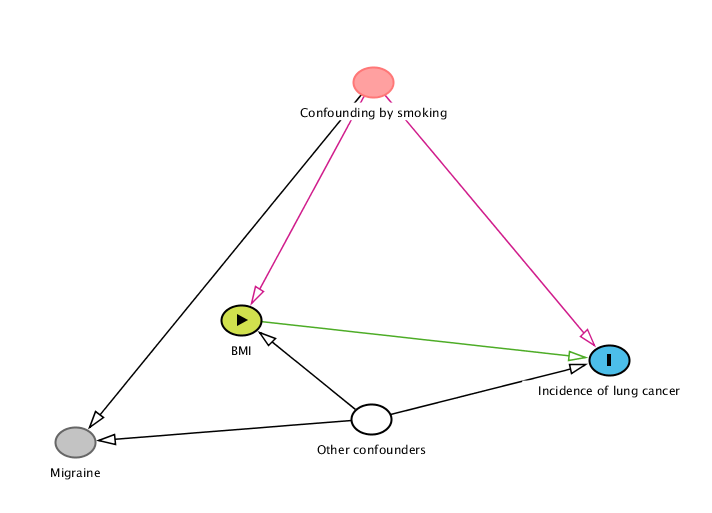


Figure legend. This DAG was created by using DAGitty V 3.0^4^ (http://www.dagitty.net/dags.html).

|  |  |  |  | Crude^1^ | | Adjusted^2^ | | Adjusted^3^ | |
| --- | --- | --- | --- | --- | --- | --- | --- | --- | --- |
| LC |  | **Cases** | **IR (per 1000 person-years)** | **HR** | **95% CI** | **HR** | **95% CI** | **HR** | **95% CI** |
| Overall | Non-migraine | 738 | 0.85 | 1.00 | Reference | 1.00 | Reference | 1.00 | Reference |
|  | Migraine | 40 | 0.47 | 0.68 | 0.49-0.93 | 0.83 | 0.60–1.14 | 0.80 | 0.58–1.11 |
| SC | Non-migraine | 110 | 0.13 | 1.00 | Reference | 1.00 | Reference | 1.00 | Reference |
|  | Migraine | 9 | 0.11 | 0.98 | 0.50–1.94 | 1.23 | 0.62–2.43 | 1.07 | 0.54–2.12 |
| AD | Non-migraine | 240 | 0.28 | 1.00 | Reference | 1.00 | Reference | 1.00 | Reference |
|  | Migraine | 17 | 0.20 | 0.85 | 0.52–1.39 | 0.99 | 0.61–1.63 | 0.93 | 0.57–1.53 |
| SQ | Non-migraine | 146 | 0.17 | 1.00 | Reference | 1.00 | Reference | 1.00 | Reference |
|  | Migraine | 9 | 0.11 | 0.81 | 0.41–1.58 | 1.01 | 0.51–1.98 | 0.95 | 0.58–1.56 |

Supplementary Table S3. Negative control using migraine as an alternative exposure to address residual confounding by smoking for the association of BMI in HUNT2 with lung cancer incidence across different lung cancer subtypes, the HUNT Study, 1995-97 to 2017 (N = 49,969)

Abbreviations: AD: adenocarcinoma; BMI: body mass index; CI: confidence interval; HR: hazard ratio; IR: incidence rate. LC: lung cancer; SC: small cell lung cancer; SQ: squamous cell lung cancer.

^1^ Age was used as the time scale in the crude model.

^2^ Adjusted for smoking [(never, former (≤10.0, 10.1-20.0 and >20.1 pack-years (pyrs)), current (≤10.0, 10.1-20.0 and >20.1 pyrs)]. Age was used as the time scale.

^3^ The Same confounders as for the association between BMI at HUNT2 and lung cancer incidence: sex, smoking, passive smoking, leisure physical activity, total sitting time daily, education, economic difficulties, family history of cancer, and self-reported chronic obstructive pulmonary disease (COPD). Age was used as the time scale.

*Tvc* option of the *stcox* command in Stata was used to model the non-proportional hazards in the adjusted^3^ model. Non-proportional hazards for LC overall: sex, smoking, and economic difficulties; for SC: economic difficulties and leisure physical activity; for AD: sex, smoking, economic difficulties, and leisure physical activity; for SQ: education.

Supplementary Table S4. Comparison of baseline characteristics of participants with complete and missing information on 75 BMI SNPs in HUNT2

| Variables | Complete information on BMI SNPs | Missing information on BMI SNPs | Whole population |
| --- | --- | --- | --- |
| Number of subjects | 54511 | 7942 | 62453 |
| Age (years), mean±SD | 49.1±16.4 | 52.3±20.0 | 49.5±16.9 |
| Number of lung cancer cases (%) | 873 (1.6) | 136 (1.7) | 1009 (1.6) |
| Female sex, % | 52.7 | 53.7 | 52.9 |
| Ever smoker, % | 55.9 | 52.2 | 55.4 |
| Ever passive smoker, % | 80.5 | 76.4 | 80.0 |
| Alcohol consumption ≥1 (times/month), % | 58.4 | 47.6 | 57 |
| Leisure physically active^1^, % | 49.0 | 41.9 | 48.1 |
| Total sitting time daily ≥8 (hours), % | 28.6 | 25.0 | 28.1 |
| Most sedentary during work, % | 23.2 | 20.3 | 22.8 |
| Education <10 (years), % | 33.5 | 38.0 | 34.1 |
| Having economic difficulties, % | 22.0 | 20.0 | 21.7 |
| Having family history of cancer, % | 25.7 | 23.2 | 25.4 |
| Having self-reported COPD, % | 2.2 | 2.6 | 2.2 |
| Having asthma, % | 5.4 | 5.7 | 5.5 |

Abbreviations: BMI: body mass index; COPD: chronic obstructive pulmonary disease; SD: standard deviation; SNPs: single-nucleotide polymorphisms.

^1^ Active: physical activity level from low to high.

Supplementary Text 2. Univariable Mendelian randomization (MR) analyses using the 61 single-nucleotide polymorphisms only for

body mass index (BMI-Only SNPs)

In the univariable MR analyses, a two-stage method was used on an externally weighted BMI genetic risk score (GRS) first. The GRS was calculated by multiplying the number of BMI-increasing alleles by the variant’s coefficient for BMI from the GIANT study and summing across the 61 BMI-Only SNPs. Using a GRS can reduce weak instrument bias by individual genetic variants and ensure that a large proportion of BMI can be accounted for and therefore increase statistical power ^5^. In the first stage of the two-stage method, BMI was regressed on GRS and a predicted value of BMI was created ^6^. The coefficient of the association between GRS and BMI was derived after adjusting for sex, age and age-squared ^7^. Based on the results of multiple testing for the correlations between the GRS and all possible confounders, self-reported COPD was suggested to be associated with the GRS with a Bonferroni corrected P value<0.005 (Supplementary Table S5). Thus, the coefficient of the association between the predicted value of BMI and incidences of LC and subtypes were derived after additionally adjusting for self-reported COPD in the second stage. The MR derived HR with 95% CI was calculated by the natural exponential function of the coefficient from the second stage. GRS based on 61 BMI-Only SNPs was suggested to explain 2.0 % of the variance in BMI in HUNT2, corresponding to a F statistics of 905 ^8^.

Additional, we used inverse-variance weighted (IVW) and MR-Egger methods based on summarized data of the 61 individual BMI-Only SNPs to test the robustness of our results. The IVW method assumes all the instruments are valid and combines the causal estimates from each genetic variant in a random effects ^9^ of the meta-analysis model ^10^. Unlike IVW method, the MR-Egger method doses not assume all the instruments are valid. Cochran’s Q tests for both IVW and MR-Egger were used to detect heterogeneity of the SNPs. The intercept term from the MR-Egger method represents an estimate of the avarage pleiotropic effect ^9^. A non-zero intercept and the P value of the intercept test <0.05 indicates the presence of horizontal pleiotropy. The outliers in IVW and MR-Egger regression methods were identifed using MR-PRESSO ^11^ method.

As the BMI SNPs that were associated with the smoking phenotypes have been excluded, the left 61 BMI-Only SNPs should be more valid instruments for BMI. The results from all the methods showed consistently positive association between genetically determined 1 kg/m^2^ increase in BMI and the incidence of adenocarcinoma (Supplementary Table S6). Cochran’s Q tests for both IVW and MR-Egger suggested heterogeneity of the SNPs (P value <0.05), but there was no clear evidence of horizontal pleiotropy since the corresponding intercepts from the MR-Egger did not deviate markedly from zero and the P value of the intercept test >0.05 (Supplementary Table S6). Based on results from the MR-PRESSO, rs2121279 was suggested as an outlier. After excluding this SNP, the heterogeneity of SNPs was largely reduced (both P value of the IVW and MR-Egger were around 0.05). No clear associations of genetically determined BMI with the incidence of lung cancer overall or with other histologic types were found.

| Variables | Coefficient^2^ | 95% CI | | P value^3^ |
| --- | --- | --- | --- | --- |
| Sex (men vs. women) | -0.08 | -0.20 | 0.05 | 0.22 |
| Smoking ^4^ (pyrs) | 0.12 | -0.02 | 0.25 | 0.10 |
| Passive smoking (ever vs. never) | 0.16 | 0.00 | 0.32 | 0.05 |
| Leisure physical activity (active^5^ vs. inactive^6^) | -0.06 | -0.22 | 0.10 | 0.43 |
| Total sitting time daily (hours) (≥8 vs. <8) | 0.05 | -0.09 | 0.19 | 0.50 |
| Education (years) (≥10 vs. <10) | -0.18 | -0.31 | -0.04 | 0.009 |
| Economic difficulties (yes vs. no) | 0.15 | -0.01 | 0.31 | 0.06 |
| Family history of cancer (yes vs. no) | -0.02 | -0.16 | 0.12 | 0.82 |
| Self-reported COPD (yes vs. no) | 0.67 | 0.25 | 1.09 | 0.002 |

Supplementary Table S5. Associations of externally weighted BMI GRS^1^ based on 61 BMI-Only SNPs with potential confounders in HUNT2, 1995-1997 (N = 54,511)

Abbreviations: BMI: body mass index; CI: confidence interval; COPD: chronic obstructive pulmonary disease; GRS: genetic risk score; SNPs: single nucleotide polymorphisms.

^1^ The externally weighted BMI GRS was calculated by multiplying the number of BMI-increasing alleles by the variant’s coefficient for BMI from the Genetic Investigation of Anthropometric Traits (GIANT) study^7^ and summing across the 61 BMI-Only genetic variants.

^2^ Coefficient was derived from linear regression for continuous variables and from logistic regression for categorized variables corresponding to 1 unit increase in GRS.

^3^ Bonferroni corrected P value was calculated as 0.05/10 =0.005.

^4^ Smoking variable [(never, former (≤10.0, 10.1-20.0 and >20.1 pack-years (pyrs)), current (≤10.0, 10.1-20.0 and >20.1 pyrs)] was regards as an ordinal (numerical) variable.

^5^ Active: physical activity level from low to high.

^6^ Inactive: no physical activity or only light physical activity ≤2 h per week.

| LC | Cases | MR methods | HRs^1^ | 95% CI | | P_Q_^2^ | P_inter_^3^ |
| --- | --- | --- | --- | --- | --- | --- | --- |
| Overall | 873 | TS^4^ | 1.04 | 0.92 | 1.18 |  |  |
|  |  | IVW | 1.05 | 0.93 | 1.19 | 0.20 |  |
|  |  | MR-Egger | 1.08 | 0.87 | 1.35 | 0.18 | 0.74 |
| SC | 136 | TS^4^ | 1.12 | 0.80 | 1.56 |  |  |
|  |  | IVW | 1.08 | 0.81 | 1.45 | 0.90 |  |
|  |  | MR-Egger | 1.23 | 0.74 | 2.06 | 0.88 | 0.56 |
| AD | 289 | TS^4^ | 1.21 | 0.98 | 1.49 |  |  |
|  |  | IVW | 1.21 | 0.95 | 1.55 | 0.005 |  |
|  |  | IVW-corrected^5^ | 1.19 | 0.95 | 1.50 | 0.05 |  |
|  |  | MR-Egger | 1.23 | 0.80 | 1.91 | 0.004 | 0.92 |
|  |  | MR-Egger-corrected^5^ | 1.32 | 0.83 | 2.08 | 0.04 | 0.56 |
| SQ | 177 | TS^4^ | 1.04 | 0.80 | 1.34 |  |  |
|  |  | IVW | 1.02 | 0.78 | 1.32 | 0.96 |  |
|  |  | MR-Egger | 0.88 | 0.56 | 1.40 | 0.96 | 0.47 |

Supplementary Table S6. The association of BMI with incidence of lung cancer overall and different histologic types based on the univariable MR analyses using 61 BMI-Only SNPs, the HUNT Study, 1995-97 to 2017 (N = 54,511)



Abbreviations: AD: adenocarcinoma; BMI: body mass index; CI: confidence interval; HR: hazard ratio; LC: lung cancer; MR: Mendelian randomization; SC: small-cell lung cancer; SNPs: single nucleotide polymorphisms; SQ: squamous cell lung cancer; TS: two-stage method; IVW: inverse variance weighted method.

^1^ Per 1 unit (kg/m^2^) increase in genetically determined BMI.

^2^ P value for Cochran’s Q test.

^3^ P value for intercept test of MR-Egger regression.

^4^ An externally weighted BMI genetic risk score (GRS) was used as instrument in the two-stage method. The coefficient of the association between GRS and BMI was derived after adjusting for sex, age and age-squared ^7^. And the coefficient of the association between the predicted value of BMI and incidences of LC and subtypes were derived after additionally adjusting for self-reported COPD.

^5^  Results from IVM and MR-Egger after excluding the outlier: rs2121279 in adenocarcinoma.

References

1.Lipsitch, M., Tchetgen, E.T. & Cohen, T. Negative controls: a tool for detecting confounding and bias in observational studies. *Epidemiology (Cambridge, Mass.)* **21**, 383 (2010).

2.Taylor, F.R. Tobacco, Nicotine, and Headache. *Headache* **55**, 1028-1044 (2015).

3.Aamodt, A., Stovner, L., Hagen, K., Bråthen, G. & Zwart, J. Headache prevalence related to smoking and alcohol use. The Head‐HUNT Study. *Eur. J. Neurol.* **13**, 1233-1238 (2006).

4.Textor, J., van der Zander, B., Gilthorpe, M.S., Liśkiewicz, M. & Ellison, G.T. Robust causal inference using directed acyclic graphs: the R package ‘dagitty’. *Int. J. Epidemiol.* **45**, 1887-1894 (2016).

5.Burgess, S. & Thompson, S.G. Use of allele scores as instrumental variables for Mendelian randomization. *Int. J. Epidemiol.* **42**, 1134-1144 (2013).

6.Angrist, J.D. & Imbens, G.W. Two-stage least squares estimation of average causal effects in models with variable treatment intensity. *Journal of the American statistical Association* **90**, 431-442 (1995).

7.Locke, A.E.et al. Genetic studies of body mass index yield new insights for obesity biology. *Nature* **518**, 197-206 (2015).

8.Sun, Y.-Q.et al. Adiposity and asthma in adults: a bidirectional Mendelian randomisation analysis of the HUNT study. *Thorax* **75**, 202-208 (2020).

9.Bowden, J., Davey Smith, G. & Burgess, S. Mendelian randomization with invalid instruments: effect estimation and bias detection through Egger regression. *Int. J. Epidemiol.* **44**, 512-525 (2015).

10.Burgess, S., Butterworth, A. & Thompson, S.G. Mendelian randomization analysis with multiple genetic variants using summarized data. *Genet. Epidemiol.* **37**, 658-665 (2013).

11.Verbanck, M., Chen, C.Y., Neale, B. & Do, R. Detection of widespread horizontal pleiotropy in causal relationships inferred from Mendelian randomization between complex traits and diseases. *Nat. Genet.* **50**, 693-698 (2018).
